# Building a neuronal infrared lens

**DOI:** 10.1186/1471-2202-12-S1-P352

**Published:** 2011-07-18

**Authors:** Roland S Utz, Alex hilgarth, Erik Jung, Leo J van Hemmen

**Affiliations:** 1 Physik Department T35, TU München, 85747 Garching, Germany; 2 Fraunhofer Institut IZM,13355 Berlin, Germany

## 

Pit vipers as well as boas and pythons possess an infrared (IR) sensitive organ. Its layout is that of a pinhole camera but with a very large pinhole. Though this way the energy flow is high, the image of the surroundings on the pit membrane turns out to be so blurred that no structures are discernible anymore for the human eye, i.e., the brain. Sichert et al. [1] have proposed an algorithm that is able to faithfully reconstruct a detailed image of the IR surroundings.

In our present work, which aims at developing a neuronal replacement of IR lenses in technical systems, we have improved the above algorithm to perform in an even better and obtain a more robust reconstruction. We have also implemented our new algorithm in an experimental setup to verify its performance. As a first stage, we have tested the proposed algorithm in an optical setup in the visual spectrum. In a next step we approach the biological ideal by modifying the measurement setup through replacing the optical camera by an infrared imaging system. The image acquisition system is based on a 160 x 120 px micro bolometer array and has allowed us to evaluate IR images within a spectral range between 7.5 and 14 µm.

In order to produce IR images of real-life scenes in a reproducible way we have developed a technique of producing “heat-radiating” objects so as to obtain a handy collection of more or less natural IR snapshots. The *KoAl-A* project tries to determine whether and how a biological system such as snake infrared vision can be mimicked by today’s micro-integration technologies.
